# Cardiovascular disease risk and prevention amongst Syrian refugees: mixed methods study of Médecins Sans Frontières programme in Jordan

**DOI:** 10.1186/s13031-017-0115-z

**Published:** 2017-07-17

**Authors:** Dylan R.J. Collins, Kiran Jobanputra, Thomas Frost, Shoaib Muhammed, Alison Ward, Abed Alrazzaq Shafei, Taissir Fardous, Sadeq Gabashneh, Carl Heneghan

**Affiliations:** 1 Nuffield Department of Primary Care Health Sciences, University of Oxford, Radcliffe Primary Care Building, Radcliffe Observatory Quarter, Woodstock Rd, Oxford, OX2 6GG United Kingdom; 2 Médecins Sans Frontières, London, United Kingdom; 3 Médecins Sans Frontières, Amman, Jordan; 4 Ministry of Health, Amman, Jordan

**Keywords:** Cardiovascular risk assessment, Cardiovascular disease, Refugee, Primary health care, Syria, Jordan, WHO PEN, World Health Organisation Package of Essential NCD Interventions for Primary Health Care in Low Resource Settings

## Abstract

**Background:**

The growing burden of non-communicable diseases (NCDs) presented new challenges for medical humanitarian aid and little was known about primary health care approaches for these diseases in humanitarian response. We aimed to evaluate Médecins Sans Frontières (MSF’s) use of total CVD risk based prevention strategies amongst Syrian refugees in northern Jordan to identify opportunities to improve total CVD risk based guidance for humanitarian settings.

**Methods:**

We evaluated CVD risk assessment and management in two outpatient NCD clinics in the Irbid governorate of Jordan using a mixed methods design with qualitative and quantitative strands of equal priority, integrated during data collection and interpretation. World Health Organisation/International Society of Hypertension (WHO/ISH) CVD risk charts requiring measured cholesterol were used in the clinics and in our analysis. An electronic database of routine clinical information was used to determine the CVD risk profile of the clinic population, the pattern and concordance of lipid-lowering treatment prescriptions, and the prevalence and accuracy of documented CVD risk scores. This was combined with semi-structured interviews with MSF health workers, which were recorded, transcribed verbatim, and analysed thematically.

**Results:**

We reviewed the clinical records of 2907 patients. One fifth (20.9%; 95% CI 19.5, 22.4) of patients had a history of CVD while 56.8% (95% CI 54.9, 58.6) of patients had a WHO/ISH risk of <10%. Only 23.3% (95% CI 21.9, 25.0) of patients had a documented WHO/ISH risk score of which 65% were correct. 60.4% (95% CI 58.6, 62.2) of patients were eligible for lipid-lowering treatment and 48.3% (95% CI 45.9, 50.6) of these patients were prescribed it. Analysis of interviews with sixteen MSF staff identified nine explanatory themes. Providers had confusion about when and how to use the risk charts, tended to favour lifestyle intervention over drug treatment, and had uncertainty about the role of lipid-lowering treatment in primary but not secondary prevention. Patients were reluctant to start, stop, or change medication and were less able to modify risk factors and benefit from health education because of their social and economic context.

**Conclusions:**

Four priority areas to improve CVD risk-based guidance for prevention in humanitarian settings include: practical training for health workers on total CVD risk assessment and associated guidance; supporting the use of CVD risk charts as a communication tool and task sharing; contextualising risk scoring in a broader, single consultation, total CVD risk-based algorithm; and targeting popular health myths amongst the community.

## Background

The growing burden of non-communicable diseases (NCDs) has presented new challenges for medical humanitarian aid and recent evidence suggested cardiovascular (CVD) morbidity and mortality increases following humanitarian disaster [1, 2]. Despite this increasing burden, little was known about the management of CVD risk in humanitarian settings and clinical guidance was urgently needed [3–5].

The humanitarian crisis in Syria, and by extension through migration southward into northern Jordan, resulted in an unprecedented burden of NCDs, borne primarily by primary care services. As of April 2016, the United Nations High Commissioner for Refugees had registered 4.8 million Syrian refugees [6], of which over 600,000 were registered in Jordan [7]. A survey of Syrian refugees households in Jordan (2014) estimated more than half had at least one member with an NCD, [8] and in 2012 almost half (46%) of all adult deaths in Syria were attributable to NCDs [9].

In 2014, Médecins Sans Frontières (MSF) started providing NCD care in two outpatient primary healthcare clinics in Northern Jordan, specifically targeting urban Syrian refugees. Since chronic disease care in Jordan was historically provided at the secondary care level, MSF developed their own total CVD risk-based guidance adapted from the World Health Organisation’s (WHO) Package of Essential NCD Interventions for Primary Health Care in Low-Resource Settings (WHO PEN) which included World Health Organisation/International Society of Hypertension (WHO/ISH) CVD risk charts requiring measured cholesterol. [10, 11]. Although the total risk approach for the prevention of CVD is widely accepted in high income countries and has been endorsed by the WHO for low- and middle-income countries, its use in humanitarian settings was unprecedented [12].

We undertook a mixed methods study of MSF’s NCD programme to evaluate the use of total CVD risk-based prevention strategies in humanitarian settings and to identify opportunities for improvement.

## Methods

### Mixed methods design

We used a mixed methods design of quantitative and qualitative strands of equal priority, integrated during data collection and interpretation. The findings of the qualitative strand were used to help explain the findings in the quantitative strand and identify opportunities for improvement.

### Quantitative methods

The quantitative strand had three objectives: (1) to determine the CVD risk profile of the clinic population; (2) to describe the pattern and concordance of lipid-lowering treatment prescriptions with guidance; and (3) to determine the prevalence and correctness of documented CVD risk scores.

We studied two MSF clinics in the Irbid governorate, Jordan, whose remit focused on providing free primary health care for urban (rather than camp-based) Syrian refugees but also for some Jordanians who required access to primary health care. The clinics accepted patients living with one of five conditions: CVD, hypertension, diabetes, chronic obstructive pulmonary disease or asthma. Patients with existing CVD (secondary prevention), diabetics aged *≥*40, patients with total cholesterol *≥*8 mmol/L, or patients with WHO/ISH risk *≥*20%, were eligible for lipid-lowering treatment based on WHO PEN [12, 13].

We adapted the inclusion criteria from WHO PEN Protocol One: all patients aged ≥40 were eligible for inclusion, in addition to adults (≥18) under 40 who smoked, were diabetic, had a family history of CVD or diabetes in a first or second degree relative, or a high waist circumference [11]. High waist circumference was defined as ≥90 cm in women and ≥100 cm in men, and smoking status was coded as positive if the patient was a current smoker or had quit in the previous 12 months, as per WHO PEN [11]. We used routinely collected patient data stored in MSF’s central NCD database. The database was managed and cleaned by MSF who routinely entered data from paper charts into the database. These data included all basic demographic information, in addition to laboratory testing results, risk factor measurements, and prescribing information on a per-visit basis. We searched this database from inception (15/12/2014) until 01/11/2015 and screened all patients for inclusion.

We conducted all analyses using the statistical software R [14]. We calculated Cohen’s kappa between documented and calculated CVD risk scores using the irr package (version 0.84) and WHO/ISH risk scores using the whoishRisk package [15, 16]. Previous history of CVD was coded as positive if the patient had a history of stable or unstable angina, myocardial infarction, angioplasty, congestive heart failure, peripheral vascular disease, any other CVD condition (e.g. atrial fibrillation), or documented cardiovascular or cerebrovascular complications.

To determine the distribution of CVD risk in the patient population, the CVD risk scores at the time of enrolment to the clinic were calculated. The index date was set to the date of enrolment and the first prospectively available systolic blood pressure and total cholesterol measurement were used to calculate the risk score.

To determine the risk score of patients prescribed lipid-lowering treatment, the index date was set to the date of first lipid-lowering treatment prescription and the first retrospectively available systolic blood pressure and total cholesterol measurement prior to the date of the lipid-lowering treatment prescription were used. For patients not prescribed lipid-lowering treatment, their risk score at enrolment was used to determine eligibility for lipid-lowering treatment.

To determine the accuracy of documented CVD risk scores, the index date was set to the date of the first documented CVD risk score and the first retrospectively available systolic blood pressure and total cholesterol measurement were used. The inter-rater reliability was calculated using Cohen’s kappa. Patients with existing CVD who had a recorded risk of ≥20% were coded as a match for the calculation of Cohen’s kappa.

We imputed missing systolic blood pressure measurements using the mean systolic blood pressure at admission (130 mmHg) because missing data were very rare (0.5%). We used a linear regression model to predict missing cholesterol values based on the complete cases dataset using three predictor variables: age, gender, and systolic blood pressure.

### Qualitative methods

The objectives of the qualitative strand were to (1) explain trends observed in the quantitative strand and (2) identify opportunities to improve total CVD risk-based guidance for humanitarian settings.

We interviewed clinical and non-clinical staff working in MSF’s NCD services in Jordan. We were primarily interested in clinicians involved directly in patient care (e.g. nurses/health promoters, pharmacists, and doctors), but also those involved in the organisation and administration of the health service. Given the unique context, we sought to interview all clinical staff, and purposively sampled non-clinical staff. Staff were notified by MSF of the project, and the interviewer (DC) described the project at staff meetings and recruited participants. At the time of the study, there were five doctors, eight nurses/health promoters, and two pharmacists employed between the two clinics. All clinical staff were Jordanian and could speak English.

After obtaining written informed consent, we conducted one-on-one, face-to-face, semi-structured interviews with participants in the workplace, but in locations that ensured privacy (e.g. office). Our interview guide was adapted from a previously published guide on a similar topic, and from our quantitative analysis [17]. The interviewer (DC) had no relationship with the interview participants before the interviews were conducted, but had conducted the quantitative analysis and was familiar with the health system in Jordan. After collecting written informed consent, 30 to 60 minute interviews were conducted. Because English was the second language of the interview participants, the interviewer adopted a technique to summarise or repeat information to clarify or confirm the meaning of statements from the participants as needed.

We recorded and transcribed interviews verbatim, and checked them for accuracy. We used NVivo 11 to code interviews. The analysis was based on the explicit meanings of the data rather than examining the underlying ideologies that shape what people say, and in this way borrowed concepts from semantic and realist approaches [18]. Analysis was primarily deductive and was guided by the fact that the purpose of the qualitative data was to help explain the quantitative findings. However, this was balanced by also allowing for an inductive approach whereby open coding could be used for the identification of relevant but unanticipated themes. A coding framework was developed and applied to all interviews independently by two authors (DC, TF), and discrepancies in coding were discussed and finalised collaboratively.

## Results

### Quantitative findings

**Fig. 1:**
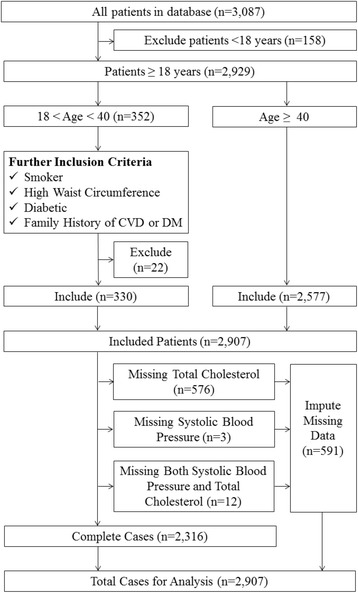
Flow chart of patients included in the quantitative strand analysis

We included 2907 of 3087 patients in the database: 158 patients aged <18 and 22 patients aged <40 did not meet the inclusion criteria (Fig. 1). Of the included cases, 591 were either missing a total cholesterol measurement (*n* = 576), a systolic blood pressure measurement (*n* = 3), or missing both measurements (*n* = 12). Missing total cholesterol was highest in those aged <40 but similar between men and women.

Table 1 reports the WHO/ISH CVD risk distribution of the included population. Most (*n* = 1650) had a CVD risk score < 10%, while one fifth (*n* = 608) had a history of CVD, placing them in a high risk category. Over half of the included population was female (61%), 71% percent of smokers were male, and two-thirds had a family history of diabetes.

**Table 1:** Prevalence of CVD risk by WHO/ISH risk category and summary of salient patient characteristics

	WHO/ISH risk category					History of CVD (*n* = 608)
	<10% (*n* = 1650)	10 to <20% (*n* = 325)	20 to <30% (*n* = 139)	30 to <40% (*n* = 70)	≥ 40% (*n* = 115)	
Percent of Total Population (95% CI)	56.8 (54.9, 58.6)	11.2 (10.1 12.4)	4.8 (4.0 5.6)	2.4 (1.9, 3.1)	4.0 (3.3, 4.7)	20.9 (19.5, 22.4)
Median Age (IQR)	50 (42–57)	64 (60–71)	66 (61.0–72.5)	64 (61.25–71)	66 (62–71)	61 (53–68)
Percent Male (95% CI)	33.8 (31.5, 36.2)	41.2 (35.9, 46.8)	41.7 (33.5, 50.4)	34.3 (23.6, 46.7)	40.9 (31.9, 50.4)	53.5 (49.4, 57.5)
Percent with type one diabetes (95% CI)	2.4 (1.7, 3.2)	0.3 (0.0, 2.0)	0.7 (0.0, 4.5)	0.0 (0.0, 6.5)	0.9 (0.0, 5.5)	0.2 (0.0, 1.1)
Percent with type two diabetes (95% CI)	42.5 (40.1, 45.0)	63.1 (57.5, 68.3)	64.7 (56.1, 72.5)	80.0 (68.4, 88.3)	80.0 (71.3, 86.70	52.3 (48.2, 56.3)
Percent who smoke (95% CI)	23.7 (21.7, 25.8)	19.7 (15.6, 24.5)	32.4 (24.8, 40.9)	28.6 (18.7, 40.8)	28.7 (20.8, 38.0)	31.2 (27.6, 35.1)
Mean SBP (mmHg) (SD)	124.39 (17.60)	139.74 (18.62)	146.58 (20.95)	156.07 (15.39)	171.64 (20.47)	129.03 (22.96)
Mean total cholesterol (mmol/L) (SD)	5.16 (0.96)	5.18 (1.13)	5.41 (1.61)	5.64 (1.25)	6.28 (1.59)	4.74 (1.11)
Percent with total cholesterol ≥8 mmol/L or (diabetes & age ≥ 40) (95% CI)	38.4 (36.1, 40.8)	63.7 (58.2, 68.9)	66.2 (57.6, 73.9)	81.4 (70.0, 89.4)	80.9 (72.3, 87.4)	52.5 (48.4, 56.5)
Percent with a family history of diabetes (95% CI)	69.0 (66.7, 71.2)	65.2 (59.7, 70.3)	48.9 (40.4, 57.5)	65.7 (53.3, 76.4)	63.5 (53.9, 72.1)	62.5 (58.5, 66.3)
Percent with a family history of premature CVD (95% CI)	39.2 (36.9, 41.6)	25.8 (21.2, 31.0)	29.5 (22.2, 37.9)	21.4 (12.9, 33.2)	29.6 (21.6, 38.9)	42.3 (38.3, 46.3)
Percent with high waist circumference (95% CI)	79.5 (77.5, 81.4)	79.7 (74.8, 83.8)	73.4 (65.1, 80.3)	85.7 (74.8, 92.6)	83.5 (75.1, 89.5)	72.7 (68.9, 76.2)

*Abbreviations*: *SBP* systolic blood pressure

Of the included patients, 60.4% (95% CI 58.6, 62.2; *n* = 1757) were eligible for lipid-lowering treatment: 95.6% (95% CI 94.5, 96.5; *n* = 1680) because they either had existing CVD (*n* = 608) or were diabetic and aged ≥40 (*n* = 1072) (Table 2). Of patients eligible for lipid-lowering treatment 48.3% (95% CI 45.9, 50.6) were prescribed treatment. Prescribing was highest amongst patients with a history of CVD and diabetics aged ≥40: 70.6% (95% CI 66.7, 74.1) and 37.4% (95% CI 34.5, 40.4), respectively. Amongst the remaining asymptomatic patients, prescribing rates were not different amongst patients above and below the treatment threshold of 20% risk: 16.7% (95% CI 9.00, 28.3) and 16.3 (95% CI 14.3, 18.6), respectively (Table 2). Of the patients with risk scores ≥20% who were prescribed lipid-lowering treatment (*n* = 11), 36.4% (95% CI 12.4, 68.4; *n* = 4) had a total cholesterol measurement immediately prior to lipid-lowering treatment prescription below 5.10 mmol/L.

**Table 2:** Lipid-lowering treatment prescribing patterns based on calculated CVD risk category, shown as mutually exclusive categories

	Secondary Prevention	Primary Prevention			
Category	History of CVD	DM & ≥40	TC ≥ 8 mmol/L	Risk <20%	Risk ≥20%
Total (*n*)	608	1072	11	1150	66
Percent prescribed lipid-lowering treatment (95% CI)	70.6 (66.7, 74.1)	37.4 (34.5, 40.4)	63.6 (31.6, 87.6)	16.3 (14.3, 18.6)	16.7 (9.00, 28.3)

*Abbreviations*: *CVD* cardiovascular disease, *DM* diabetes mellitus, *TC* total cholesterol

Only 23.3% (95% CI 21.9, 25.0; *n* = 680) of patients had a documented risk score, and nearly all (*n* = 655) were recorded as low-risk (i.e. WHO/ISH risk <20%) (Table 3). Agreement between documented risk and calculated risk was poor (Cohen’s kappa 0.178, *p* < 0.05). Of the 25 patients with a documented high risk score (i.e. WHO/ISH risk ≥20%), 6 (24%, 95% CI 0.09, 0.45) were actually low risk, whilst 130 (20%, 95% CI 0.17, 0.23) of the documented low risk patients were actually high risk (Table 4).

**Table 3:** Agreement between documented and calculated WHO/ISH CVD risk scores

		Calculated WHO/ISH CVD Risk Score					History of CVD	Total
		<10%	10 to <20%	20 to <30%	30 to <40%	≥ 40%		
Documented WHO/ISH CVD Risk Score	<10%	413	52	25	15	14	56	575
	10 to <20%	38	22	10	3	2	5	80
	20 to <30%	2	2	4	2	3	1	14
	30 to <40%	1	1	0	4	4	0	10
	≥ 40%	0	0	0	0	1	0	1
	Total	454	77	39	24	24	62	680

**Table 4:** Agreement between documented and calculated WHO/ISH CVD after aggregating by the clinically significant threshold of WHO/ISH risk 20%, where individuals with a history of CVD are categorised as high risk

		Calculated WHO/ISH CVD Risk Score		
		≥ 20% (High)	<20% (Low)	Total
Documented WHO/ISH CVD Risk Score	≥ 20% (High)	19	6	25
	<20% (Low)	130	525	655
	Total	149	531	680

### Qualitative findings

**Fig. 2:**
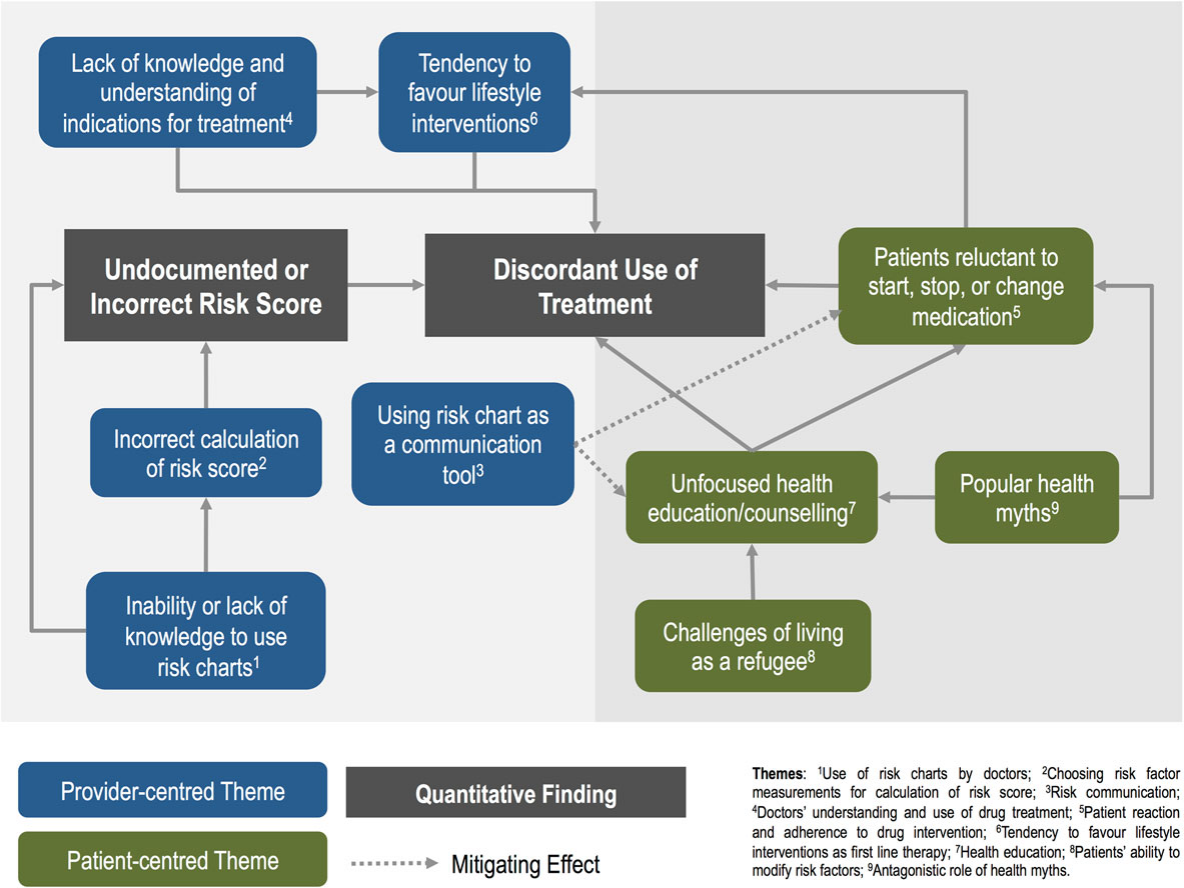
Integration map of qualitative and quantitative strands illustrating the relationships between qualitative themes and with the main quantitative findings

We interviewed 16 participants: five were doctors; seven were nurses, and the remaining a mix of health promoters, pharmacists, and managers. The doctors had a range of past work experience and postgraduate education; one had completed a family medicine residency, whilst the rest had only partially completed or not completed postgraduate training. The results of the thematic analysis with supporting quotations are summarised in Table 5, and the relation of the themes to each other and the quantitative findings are mapped in Fig. 2. Nine themes were identified which are broadly grouped into provider-centred themes and patient-centred themes.

**Table 5:** Summary of qualitative findings with example quotations

	Theme	Summary	Quotation(s)
Provider-Centred Themes	Use of risk charts by doctors	Although some doctors used the charts correctly, there was generally confusion about when to use the charts, and an inability to calculate a risk score during the first consultation without cholesterol information	*“Yes, they are 90 years old. When I look at them, “Okay, we will not do the risk assessment for this patient”. Because I don’t know how.” – Doctor D* *“So if it’s the first time the patient is coming I need to wait for the labs to calculate what the percentage is.” – Doctor A* *“I think I’m using it with most of patients because the score gives you a clear idea about the risk and how to react so I depend on it a lot.”– Doctor B*
	Choosing risk factor measurements for calculation of risk score	Diverse and incorrect methods used when recording and choosing risk factor measurements for the calculation of a risk score	“*most of the time I use the highest [reading] to be in the safe side.” – Doctor B* *“The lower reading I take it.” – Nurse A* *“Some patients when we measure blood pressure it is 210. At that visit they are 210/120 something like that, so I can’t do it. I postpone the risk assessment to the next visit when we have better reading.”– Doctor D*
	Tendency to favour lifestyle interventions as first line therapy	Doctors tended to favour lifestyle intervention over drug intervention even in patients where drug intervention was indicated	*“[if] the cardiovascular risk is 20–30 [%], we can decrease it by normalization of blood pressure that’s high and the cholesterol level if it’s high, we can give him 3 months to 4 months diet and then recheck it. If it’s still high we can start statin to reduce it.” – Doctor C* *“Yes, so do the first line and then pharmacological treatment.” – Doctor E* *“The patient in the first maybe refuse to take the statin. We give him a chance for 3 month to change the lifestyle, to change about diet, about their physical activity.” – Nurse D*
	Doctors’ understanding and use of drug treatment	Doctors had a good understanding of the use of lipid-lowering treatment in secondary prevention but some weren’t certain about the role of treatment in primary prevention	*“Nowadays, up till now, the studies said that there’s no role for statin as primary prevention that’s what I know. So no, I don’t start statin, if the cholesterol level is normal, as a primary prevention.”– Doctor B* *“Yes, if the patient having for example a high risk and previously they’re having ischaemic heart disease or for example peripheral arterial disease, we should prescribe statin even if [cholesterol] is normal.”– Doctor E*
	Risk Communication	Risk charts were used by doctors to help communicate with patients and make decisions; nurses did not use CVD risk charts, but felt they could be helpful during counselling sessions.	*“So we are just, I mean showing indication that you are in the green area, [...] so you don’t need to take aspirin because you might have side effects more than the benefits from aspirin and actually a lot of them they are convinced [...]. So it is very helpful and it is convincing.”– Doctor E* *“From my experience the colours are best for our patient from numbers.”* *– Nurse E* *“When we simplify this [CVD risk chart] for them, they will do just the same. They will drop it and they will start saying, “Okay. I am 50. I was in the dark red here because my blood pressure was 180 or more so I was in the dark red zone. Now because I quit smoking, I’m here in the orange zone, for example, in the yellow zone. And after that, okay, my blood cholesterol dropped from eight to five.” So they can just put their own thoughts and as you know, the colour is really useful.”* *– Nurse C*
Patient-Centred Themes	Patient reaction and adherence to drug intervention	Patients were reluctant to start, stop, or change medication and were often not adherent	*“We tell them that I like to add statin a new medication that’s called statin, it’s for cholesterol. And they usually react, “I don’t have any cholesterol and no I don’t have any problems with my cholesterol,” or something like that.” – Doctor A* *“Many patients when you give a new medication they just, “No”” – Doctor C* *“Most of them they don’t have problem with it. A small percentage they don’t want to change their medication…and they are not convinced.”– Doctor E* *“Why didn’t you use it? “I didn’t like it, I didn’t feel well,” or, “I felt my blood pressure was down, I didn’t [have] headache, so I didn’t take my drugs.”” – Person C*
	Antagonistic role of health myths	Many health myths existed amongst the Syrian community and these myths can antagonise the advice of clinicians	*““If I drink a cup of water plus one tablespoon of vinegar, it’s bad on the cholesterol?” This is a common question.” // “It’s something usually in Facebook.”– Person A* *“They are taking an aspirin because they are told that every patient above 40 must take aspirin as prophylactic, yes. They are told not by doctor by neighbours by relatives, yes.” – Doctor C* *“Sometimes they accept the neighbours’ opinion more than us.” – Nurse A* *“Yes. Many of our women didn’t want to follow diet regimen or DM diet… they want medication to lose weight and they ask about medication.” // “I told them that it’s not useful…but many of them search about them and bring and use.” – Nurse E*
	Patients’ ability to modify risk factors	Security concerns, socio-economic deprivation, shame, and stress can reduce the ability of patients to adhere to modify their risk factors	*“they have a fear of walking outside because they didn’t have an ID or an UNHCR paper so they refuse walking.” – Nurse E* *“Some patients gradually decrease their cigarettes, but suddenly when they come “I increased my consumption.” “Why?” “Because my brother left away in Syria, because my son died, because I need more money.” – Nurse B* *“Sometimes our patients are shamed to tell you about that. Just you hear “you must take vegetables, fruit just one time a week.” They are still silent, because sometimes they do not have anything. That is the problem. It is better, I think, in the home visit to give a good picture or clear picture” – Nurse F*
	Health education	Individual health education sessions were often co-opted by more immediate needs of patients; education in a group was seen as more effective	*“They’re just continually thinking about that their son was killed and that’s all they can think about…. And then so then the health education portion gets pushed aside because they need to sit and just listen to the patient.” – Person B* *“They just keep telling us about what happened in Syria and after they finish, we start our health coaching sessions.” – Nurse C* *““I think the refugees become more relax when they talk together, see same cases like, “I’m not the only one. I have cardio or I have DM there’s a lot of person like me.” It’s more unique. I think it’s more effective than the individual session.”– Person A*

### Provider-centred themes

#### Use of risk charts by doctors

Doctors had a limited understanding of total CVD risk, which affected their ability to use the risk charts. One doctor routinely used risk charts with patients; others, however, did not understand who was eligible for risk assessment and used it in an ad hoc manner. One doctor misunderstood how to categorise risk factor values and thought that patients with extreme values (e.g. age > 70 or SBP >180) could not be risk assessed. A barrier that affected all doctors was the need for a laboratory requisition to obtain total cholesterol information before risk assessment, thereby delaying the use of the risk charts to a follow-up visit.

#### Choosing risk factor measurements for calculation of risk score

We identified four approaches that doctors used to choose SBP and cholesterol values to calculate a risk score. These consisted of using the most recent value, using the highest value, calculating multiple scores with multiple risk factor values, or delaying the calculation to the next visit when the readings were more reasonable.

#### Tendency to favour lifestyle interventions as first line therapy

According to MSF guidance, patients with total cardiovascular risk of 20% or more were eligible for lipid-lowering treatment. However, many doctors preferred to use lifestyle interventions alone as first-line treatment, even in high risk patients eligible for lipid-lowering treatment.

#### Risk communication

Doctors often used risk charts as a communication tool with patients, as it helped avoid the use of numbers. Doctors felt that communicating to a patient that they were high-risk tended to induce fear, which motivated them to reduce their own risk factors. Communicating low-risk scores to patients was sometimes helpful when de-prescribing because it reassured patients. This reassurance was also at times counterproductive by encouraging apathy, so in these instances doctors avoided communicating the risk score. Nurses and health promoters were not expected to use cardiovascular risk charts, and they did not, but they did use colour-coded risk charts with patients for blood pressure and HbA1c that they found very useful. These professionals saw a role for using the CVD risk charts in their work and some nurses had a good understanding total CVD risk.

#### Doctors’ understanding and use of drug treatment

Reliance on lifestyle intervention and discordant use of lipid-lowering treatment might also be explained by the limited understanding of the doctors on the use of treatment in primary prevention. Doctors often used a single risk factor approach, rather than a total risk approach. All doctors understood that lipid-lowering treatment lowers cholesterol; however, some were confused about its role in risk reduction when cholesterol levels in high risk individuals were ‘normal.’ Most doctors understood the role of lipid-lowering treatment in secondary prevention.

### Patient-centred themes

#### Patient reaction and adherence to drug intervention

Such reliance on lifestyle interventions as first line therapy might be explained in part by patient preferences. Patients were reluctant to start, stop, or change medication and were often not adherent. For example, some high risk patients refused treatment when their cholesterol levels were ‘normal.’ At enrolment to the clinic some patients were already taking medication, and were often reluctant to change or stop medications. The migration patterns of some refugees also affected adherence because they were unable obtain medication during trips to Syria.

#### Health education

Given that many doctors used lifestyle interventions as first line therapy, health education by nurses and health promoters was a core component of the clinical care. These sessions were often directed by the patients’ more immediate needs, such as psychological or emotional distress, and therefore the health education objectives were not met. One nurse noted that because of the stressful environment, it sometimes took three sessions with a patient until rapport was established and patients start to openly discuss lifestyle changes. Despite these challenges, most clinicians felt that many patients were able to make positive changes. Health education was also conducted with groups of patients and was seen as more effective than individual sessions because patients were more relaxed and could share experiences with their peers.

#### Patients’ ability to modify risk factors

Despite intensive and dedicated time for health education, many patients were not interested or able to exercise because of personal security concerns, stress, and psychological distress. Women were seen to face greater challenges to exercise, because of security and sometimes cultural restraints of exercising outdoors. Many patients were embarrassed to fully disclose their psycho, social, or occupational context, and therefore the recommendations of clinicians were sometimes unrealistic.

#### Antagonistic role of health myths

Health myths – popular but untrue anecdotes about health – were prevalent in the patient population. Facebook was noted as a medium for sharing myths, including drinking vinegar to reduce cholesterol. Many of the health claims antagonised the goals of clinicians, but could be popular because some people were sceptical of their healthcare providers and trusted their peers.

## Discussion

We conducted a mixed methods study MSF’s NCD program for Syrian refugees in Jordan. The quantitative strand included 2907 patient records and was combined with individual interviews of 16 MSF health workers. This demonstrated that despite implementation of total CVD risk-based guidance, few patients had a documented and correct CVD risk score, and half of high risk patients were not prescribed lipid-lowering treatment. Many of the risk scores document in patient records were inaccurate; of patients with a documented low risk score, one in five were truly high risk. The qualitative analysis found nine themes that together helped theorise the quantitative findings and identify opportunities to improve the use of total CVD risk-based approaches in humanitarian settings.

The low prevalence and accuracy of documented risk scores may partially explain the discordant use of treatment but the qualitative strand also helped explain the quantitative findings. We found a tension between the need to use drug intervention for primary prevention and the tendency of doctors to prefer lifestyle interventions without drug intervention. This may be explained by a misunderstanding of the role of treatment in primary prevention by the doctors, especially amongst individuals with high risk but normal cholesterol, but also because some patients were reluctant to start new medication and were influenced by health myths. This became problematic because individual health counselling sessions were often co-opted by psycho-social counselling rather than lifestyle education, resulting in patients not adhering to lifestyle interventions and not on treatment. Furthermore, the risk assessment workflow was laboratory-dependent, which meant that doctors tended to defer use of the risk charts until the second visit when the test result would normally be available. Since follow-up visits were typically 15 min, we speculate that risk assessment would be forgotten or overlooked. Such an emphasis on risk scoring may have also distracted clinicians from more simple risk assessment – 30% of patients with existing CVD and 60% of diabetic patients over 40 remained untreated. These findings are consistent with findings of larger studies in Europe which show that many secondary prevention patients do not achieve sufficient risk factor control [19].

There was significant variability between clinicians in the way that risk charts and clinical guidelines were used; this was sometimes the result of misunderstandings of how to calculate a risk score and which risk factor measurements to use, indicating a need for further training and simplification. This has been observed in other jurisdictions implementing CVD risk scoring, and may help explain the low accuracy and prevalence of documented risk scores [17, 20, 21].

### Implications for policy and practice

Integration of the quantitative and qualitative findings identified four priority areas to improve total CVD risk-based guidance and prevention in humanitarian settings.

First, our findings are consistent with others showing that the implementation of guidelines alone is not effective at changing practice [22] and recent systematic review evidence show that health care provider education is an important component of improving adherence to CVD guidelines [23]. Given the lack of familiarity of the health care providers with CVD risk scoring, education should include detailed practical exercises on calculating risk scores and measurement of risk factors.

Second, there is potential for greater integration of a total CVD risk approach with the role of nurses and health promoters. Although there is limited evidence on task sharing for CVD management in LMIC, [24] the WHO has recently published guidance on task sharing for total CVD risk assessment in low-resource settings, [25] and there is evidence to support the role of non-physician health workers conducting CVD risk assessments [26].

Third, risk scoring should be contextualised in a broader risk assessment algorithm that can be conducted within a single consultation that reinforces the identification of patients with existing disease and diabetics over the age of 40. Risk scores without measured cholesterol, such as those published by WHO, are less complex and allow single consultation risk scoring [10]. Those in charge of implementation must determine whether to optimise adherence to simple clinical protocols before adding additional complexity, such as cholesterol testing, with potentially marginal returns for patient outcomes [27].

Fourth, greater engagement with patients in the organisation and planning of care may help build trust between the community and health care provider. Although further research is needed for its use in this context, facilitated participatory learning and action may help engage the community in identifying and correcting health myths and misconceptions [28, 29]. Since Facebook was an important vector of health myths, social media should be closely considered for corrective health promotion.

### Strengths and limitations

To our knowledge we are the first to report findings from the use of total CVD risk assessment in humanitarian settings. This work was strengthened by its large size and mixed method design. Our findings highlight important insights to the use of total CVD risk approaches in humanitarian settings, and although valuable to a wide audience, are most directly generalizable to the Eastern Mediterranean region. As the management of NCDs in primary health care expands, our study should be replicated in similar settings, and researchers may consider also assessing the prescription of blood pressure-lowering treatments and aspirin. Whilst the qualitative strand helped theorise factors relating to adherence, we were unable to measure adherence in the quantitative strand, and prescribing rates should not be interpreted as synonymous with adherence. Since the patient population study was recruited for care because of their increased risk of NCDs, their risk factor levels should not be generalized to the entire Syrian refugee population in the Eastern Mediterranean. We were unable to determine quantitatively the proportion of patients who were offered but refused treatment, which may have underestimated prescribing concordance. It is also possible that some clinicians were calculating but not documenting CVD risk scores, despite a dedicated space for CVD risk scores in the patient record. We did not assess differences by gender, not least because of the sample size and ratio of men to women.

## Conclusions

A total CVD risk approach for the management of CVD in primary health care should be simplified toward a model that can be used in a single consultation and clearly contextualises the role of risk scoring in a broader risk assessment algorithm, emphasising secondary prevention and the identification of older diabetic patients. Training of health staff on a total cardiovascular risk approach and context-specific patient considerations, such as the role of health myths, the increased need for building rapport with patients, and the psycho-social-occupational context of patients is likely to be necessary to enable effective implementation.

## Abbreviations

CVD: *Cardiovascular Disease*
MSF: *Médecins Sans Frontières*
NCD: *Non-Communicable Disease*
SBP: *Systolic Blood Pressure*
WHO: *World Health Organisation*
WHO/ISH: *World Health Organisation/International Society of Hypertension*
WHO PEN: *World Health Organisation Package of Essential NCD Interventions for Primary Health Care in Low Resource Settings*
